# Placental transcriptomic signatures of prenatal and preconceptional maternal stress

**DOI:** 10.1038/s41380-023-02403-6

**Published:** 2024-01-11

**Authors:** Brennan H. Baker, Sophie Freije, James W. MacDonald, Theo K. Bammler, Ciara Benson, Kecia N. Carroll, Daniel A. Enquobahrie, Catherine J. Karr, Kaja Z. LeWinn, Qi Zhao, Nicole R. Bush, Sheela Sathyanarayana, Alison G. Paquette

**Affiliations:** 1 University of Washington, Seattle, WA USA; 2 Seattle Children’s Research Institute, Seattle, WA USA; 3 Global Alliance to Prevent Preterm Birth and Stillbirth (GAPPS), Lynnwood, WA USA; 4 Icahn School of Medicine at Mount Sinai, New York, NY USA; 5 University of California San Francisco, San Francisco, CA USA; 6 University of Tennessee Health Sciences Center, Memphis, TN USA

**Keywords:** Predictive markers, Psychology

## Abstract

Prenatal exposure to maternal psychological stress is associated with increased risk for adverse birth and child health outcomes. Accumulating evidence suggests that preconceptional maternal stress may also be transmitted intergenerationally to negatively impact offspring. However, understanding of mechanisms linking these exposures to offspring outcomes, particularly those related to placenta, is limited. Using RNA sequencing, we identified placental transcriptomic signatures associated with maternal prenatal stressful life events (SLEs) and childhood traumatic events (CTEs) in 1 029 mother-child pairs in two birth cohorts from Washington state and Memphis, Tennessee. We evaluated individual gene-SLE/CTE associations and performed an ensemble of gene set enrichment analyses combing across 11 popular enrichment methods. Higher number of prenatal SLEs was significantly (FDR < 0.05) associated with increased expression of *ADGRG6*, a placental tissue-specific gene critical in placental remodeling, and decreased expression of *RAB11FIP3*, an endocytosis and endocytic recycling gene, and *SMYD5*, a histone methyltransferase. Prenatal SLEs and maternal CTEs were associated with gene sets related to several biological pathways, including upregulation of protein processing in the endoplasmic reticulum, protein secretion, and ubiquitin mediated proteolysis, and down regulation of ribosome, epithelial mesenchymal transition, DNA repair, MYC targets, and amino acid-related pathways. The directional associations in these pathways corroborate prior non-transcriptomic mechanistic studies of psychological stress and mental health disorders, and have previously been implicated in pregnancy complications and adverse birth outcomes. Accordingly, our findings suggest that maternal exposure to psychosocial stressors during pregnancy as well as the mother’s childhood may disrupt placental function, which may ultimately contribute to adverse pregnancy, birth, and child health outcomes.

## Introduction

Maternal stress before and during pregnancy has emerged as a major contributor to long term offspring health based on the Developmental Origins of Health and Disease (DOHaD) premise [1, 2]. Recent meta-analyses suggest the importance of maternal prenatal stress for offspring health across a range of outcomes including birth weight [3, 4], obesity [5–7], asthma [8, 9], mental health [10, 11], and neurodevelopmental disorders such as autism spectrum disorder and attention-deficit hyperactivity disorder [12]. However, the mechanisms linking prenatal stress to child development are not fully understood. Evidence suggests mediating roles of the neuroendocrine stress system, immune system, gut microbiome, and telomere biology [13, 14], although more mechanistic research is critically needed in tissues that play functional roles in the maternal-fetal interface. Improved mechanistic insight may support the development of interventions and open up new avenues in precision medicine aimed at reducing the burden of maternal stress-associated disease.

As the regulator of nutrient, waste, and gas exchange between the mother and fetus, the placenta is recognized as an essential functional link between the maternal environment and fetal programming [15, 16]. For instance, phenotypes related to the efficiency of placental transfer, including weight, thickness, surface area, and the ratio of placental to fetal weight have been linked to both maternal nutrition [17, 18] and offspring coronary heart disease [19, 20]. In addition to structural adjustments in response to the maternal environment, the placenta may also go through changes at the molecular level by altering gene expression. Because gene expression is cell type and tissue specific, it is essential to quantify the transcriptome in the most relevant, functional tissues in relation to a study question. Given its unique role in the maternal-fetal interface, the placenta is the ideal target tissue for the study of prenatal exposures. Moreover, studies show that the placental transcriptome and epigenome may respond to maternal exposures including particulate matter [21, 22], phthalates [23, 24], and stress [25, 26]. Investigating placental gene expression and epigenetic changes associated with maternal stress during pregnancy may help uncover mechanisms linking prenatal stress with child development [23].

Although DNA methylation differences associated with maternal stress have been examined [26, 27], the literature investigating transcriptomic alterations remains limited. Epigenomic and transcriptomic analyses generate distinct, complementary information, but the transcriptome may provide better functional insights by capturing all RNA molecules transcribed from genes that are actively expressed. The majority of studies investigating differential gene expression associated with maternal stress have utilized targeted approaches, which limit examination to just a handful of candidate genes [28–30]. One study profiled the placental transcriptomic signatures in 131 mother-child dyads associated with pregnancy during the environmental stressor of living through Hurricane Sandy [25]. In addition to identifying 221 differentially expressed genes associated with pregnancy during that natural disaster, Nomura et al. uncovered genes that mediated associations between this maternal stress exposure with child aggression and anxiety [25]. To more comprehensively explore gene expression differences associated with prenatal stress, larger, better powered transcriptomic studies, and studies of more commonly experienced stressors not restricted to natural disaster-associated stress, are needed.

DOHaD approaches have expanded to encompass a wider window during which exposures may affect development. In addition to maternal stress during pregnancy, emerging evidence suggests that stressful experiences during the mother’s earlier developmental periods may confer changes to her biology that later affect the development of her child [10, 31]. Compared with prenatal stress, the mechanisms of intergenerational transmission of the effects of preconceptional stress are less understood. Very few studies have investigated whether the effects of preconceptional traumatic experiences may be transmitted to later generations via changes in gene expression or DNA methylation [32, 33]. One of the first studies examining the transmission of preconceptional trauma found lower blood FKBP prolyl isomerase 5 (*FKBP5*) methylation in the children of Holocaust survivors compared to demographically comparable controls [34]. Placental DNA methylation in key regulatory regions of this gene is associated with both placental *FKBP5* gene expression and infant neurodevelopmental outcomes [35]. Taken together, this work illustrates how placental molecular biology may serve as an intermediate between preconceptional stress and child development. Another targeted approach found sex-specific associations of maternal childhood trauma with gene expression and methylation of *BDNF* in cord blood [36]. Genome-wide approaches are even rarer. One study examined child genome-wide DNA methylation patterns in saliva associated with a maternal trauma measure designed to screen for potentially traumatic events over the respondent’s lifetime (Life Events Checklist) [33]. Another study examined placental genome-wide DNA methylation differences associated with socioeconomic adversity, framed as a chronic source of stress throughout the mother’s life [26]. However, to the best of our knowledge, there are no transcriptome-wide association studies simultaneously examining and distinguishing between prenatal and preconceptional maternal stress and the transcriptome of any tissue, including the placenta.

The primary objectives of this study were to examine associations of maternal exposure to stressors during pregnancy and her own childhood with the transcriptome of her infant’s placenta. Accumulating evidence indicates fetal sex differences in developmental vulnerability to prenatal stress [37]. We have also observed that fetal sex is a modifier of the relationship between other prenatal exposures and the placental transcriptome [23, 38]. Thus, we further hypothesized that associations of intergenerational stress with placental gene expression may be sex-specific. This study addresses several limitations of prior transcriptomic studies of prenatal stress, namely, small sample sizes, targeted gene approaches, lack of transcriptome-wide analyses of preconceptional maternal stress, and focus on natural disaster-related stress, which may not be generalizable to other sources of stress such as socioeconomic risk and family violence.

## Methods

### Study population

The ECHO prenatal and early childhood pathways to health (ECHO-PATHWAYS) consortium, described previously [39], is a study that harmonizes extant data and develops new data collection protocols for three pregnancy cohorts from diverse populations across the country. The consortium’s core aim is to explore the impact of chemical exposures and psychosocial stressors experienced by the mother during pregnancy on offspring growth and child development, and to assess potential underlying placental mechanisms. This analysis includes data from two of the consortium’s cohort studies in which placental transcriptomics data are available: the Conditions Affecting Neurocognitive Development and Learning in Early Childhood (CANDLE) study [40], and a subset of the Global Alliance to Prevent Prematurity and Stillbirth (GAPPS) study sample that has been enrolled in ECHO-PATHWAYS (GAPPS-PW) after their children were born.

CANDLE enrolled 1 503 women from Shelby County/Memphis, Tennessee, from 2006 to 2011 during the second trimester of pregnancy. Briefly, women were considered eligible if they were Shelby County, TN residents, between 16 and 40 years of age, had singleton pregnancies without complications at enrollment, and planned to deliver at a participating study hospital. Participants aged 18 years or older provided informed consent, while assent and consent from legally authorized representatives were obtained for those under 18 years prior to enrollment.

GAPPS was launched by the Seattle Children’s Hospital in 2007 to study the impact of adverse birth outcomes. In 2017, eligible participants were re-contacted to participate in the ECHO-PATHWAYS study. More than 600 mothers and children have been re-enrolled, and recruitment is still ongoing. Eligibility criteria included delivery in Seattle, WA (Swedish Medical Center) or Yakima, WA (Yakima Valley Memorial Hospital), availability of at least one pregnancy urine sample, initial GAPPS enrollment and completion of questionnaire, and GAPPS child currently 4–7 years of age. Study protocols were approved by the Institutional Review Boards of the University of Tennessee Health Science Center for CANDLE, the Seattle Children’s Research Institute for GAPPS, and the University of Washington for ECHO-PATHWAYS.

**Fig. 1:**
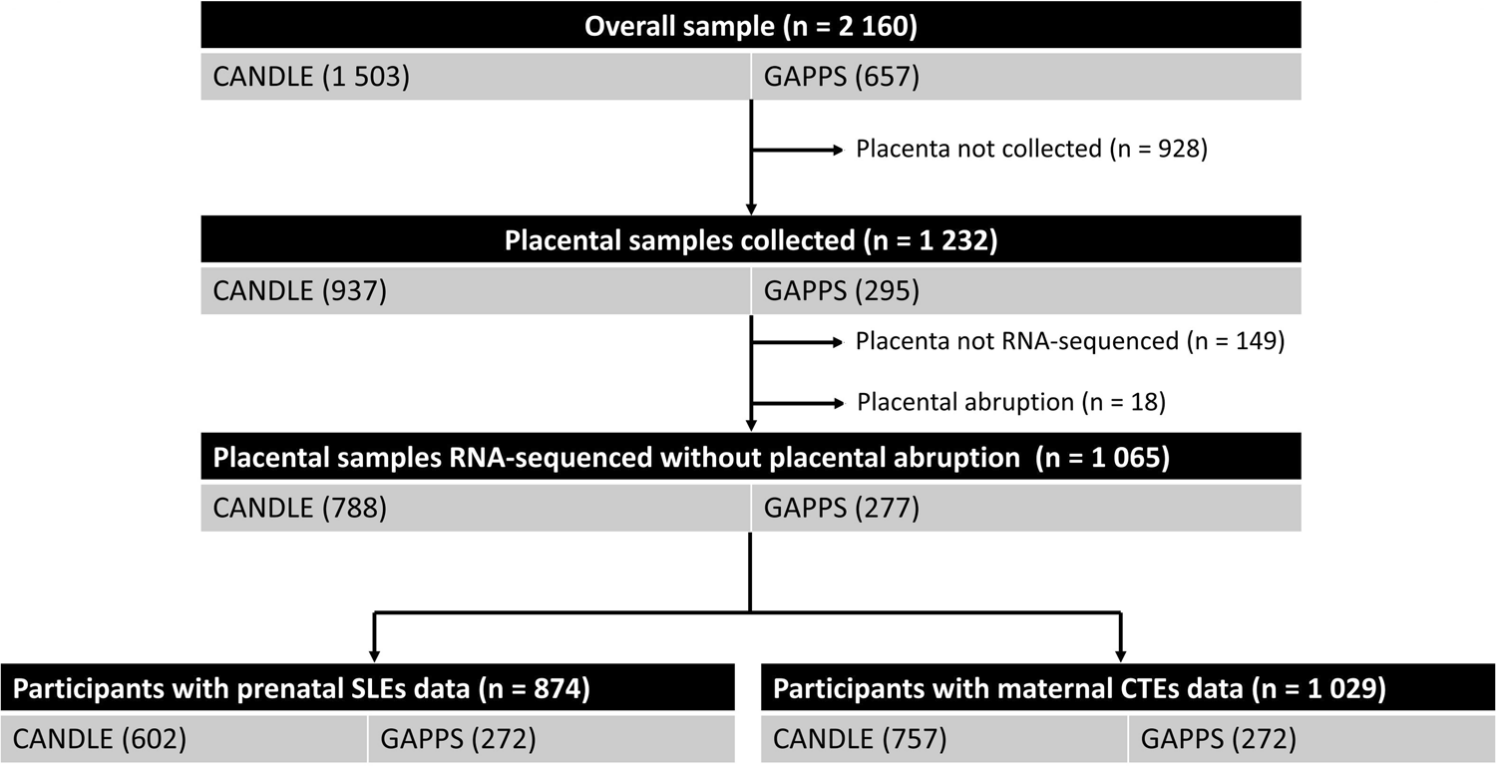
Inclusion criteria flowchart. Study flowchart for inclusion of participants shows analytic sample sizes remaining from the implementation of each exclusion criterion. Participants come from two US pregnancy cohorts in the ECHO-PATHWAYS Consortium: CANDLE (Conditions Affecting Neurocognitive Development and Learning in Early Childhood) and GAPPS (Global Alliance to Prevent Prematurity and Stillbirth).

An inclusion criteria flowchart is presented in Fig. 1. Briefly, from the CANDLE (*n* = 1 503) and GAPPS (*n* = 657) cohorts, the ECHO-PATHWAYS consortium collected and conducted RNA-sequencing on a subset of placentas (*n* = 794 from CANDLE and *n* = 289 from GAPPS). After removing participants with missing placental transcriptomics data and placental abruption (*n* = 18), our study included 1 065 participants, among whom 1 029 had complete CTE data and 874 had complete SLE data (Fig. 1).

### Exposures

Objective maternal stress exposures were retrospectively evaluated during postnatal follow up assessments (age 4–6 for GAPPS, age 8 for CANDLE). Exposures to prenatal Stressful Life Events (SLEs) were assessed using a survey adapted from the Centers for Disease Control and Prevention Pregnancy Risk Assessment Monitoring System (PRAMS) [41]. Women were asked to respond yes or no to 14 statements covering multiple types of stressful life events they experienced during pregnancy, such as those relating to relationship problems, housing or financial issues, legal problems, and illness or death of a loved one (Supplementary Table 1). Maternal childhood trauma exposures (CTEs) were assessed via responses to three questions from the Traumatic Life Events Questionnaire [42]. In this questionnaire, women reported whether they were physically abused before age 18 years, witnessed family violence before age 18 years, or experienced sexual abuse before age 13 years. Affirmative responses were summed to produce scores for both prenatal SLEs (range = 0-14) and maternal CTEs (range = 0-3), where higher scores indicate more SLEs experienced during pregnancy and more traumatic exposures experienced during childhood, respectively. Prior studies have shown consistency in the retrospective report of exposure to traumatic events during childhood [43] and stressful life experiences during pregnancy [44].

### Placental transcriptomics

The ECHO PATHWAYS consortium sequenced a subset of placentas from each cohort (*n* = 289 from GAPPS and *n* = 794 from CANDLE). Placental tissue sampling, RNA isolation, and RNA sequencing have previously been described for CANDLE and GAPPS participants [21, 23]. In the CANDLE study, within 15 min of delivery, a piece of placental villous tissue in the shape of a rectangular prism with approximate dimensions of 2×0.5×0.5 cm was dissected from the placental parenchyma and cut into four ∼0.5-cm cubes. The tissue cubes were placed in a 50-mL tube with 20 mL of RNAlater and refrigerated at 4°C overnight ( ≥ 8 h but≤24 h). Each tissue cube was transferred to an individual 1.8-mL cryovial containing fresh RNAlater. The cryovials were stored at −80°C, and the fetal villous tissue was manually dissected and cleared of maternal decidua. Following dissection, the fetal samples were placed into RNAlater and stored at −80°C.

In the GAPPS study, within 30 min of delivery, 8 mm full-thickness vertical tissue punches from the placental disc were taken and put into 5 ml tubes containing approximately 3 ml of RNAlater and stored at −20 °C before specimens were shipped to the GAPPS facility. Samples were then stored at −20 °C. Punches were thawed and maternal decidua was removed, and the fetal-side of the placental punch was cut-off from the rest of the punch, and divided into 1–3 pieces with mass ranging from 10 mg to 30 mg. Each sample was placed in 1 ml RNAlater and stored at −20 °C until shipment for further processing.

For both cohorts, approximately 30 mg of fetal villous placental tissue was used for RNA isolation. The tissue was homogenized in tubes containing 600 μL of Buffer RLT Plus with β-mercaptoethanol using a TissueLyser LT instrument (Qiagen, Germantown, MD). RNA was isolated using the AllPrep DNA/RNA/miRNA Universal Kit (Qiagen) according to the manufacturer’s recommended protocol. RNA purity was assessed by measuring optical density ratios (OD_260/230_ and OD_280/260_) with a NanoDrop 8000 spectrophotometer (Thermo Fischer Scientific, Waltham MA). RNA integrity was determined with a Bioanalyzer 2100 using RNA 6000 Nanochips (Agilent, Santa Clara, CA). Only RNA samples with an RNA integrity number (RIN) of >7 were sequenced.

All RNA sequencing was performed at the University of Washington Northwest Genomics Center. Total RNA was poly-A enriched to remove ribosomal RNA, and cDNA libraries were prepared from 1 μg of total RNA using the TruSeq Stranded mRNA kit (Illumina, San Diego, CA) and the Sciclone NGSx Workstation (Perkin Elmer, Waltham, MA). Each library was uniquely barcoded and subsequently amplified using a total of 13 cycles of PCR. Library concentrations were quantified using Qubit Quant-it dsDNA High Sensitivity assay fluorometric quantitation (Life Technologies, Carlsbad, CA). Average fragment size and overall quality were evaluated with the DNA1000 assay on an Agilent 2100 Bioanalyzer. Each library was sequenced to an approximate depth of 30 million reads on an Illumina HiSeq sequencer. RNA sequencing quality control was performed using both the FASTX-toolkit (v0.0.13) and FastQC (v0.11.2) [45]. Transcript abundances were estimated by aligning to the GRCh38 transcriptome (Gencode v33) using Kallisto [46], then collapsed to the gene level using the Bioconductor tximport package, scaling to the average transcript length [47].

### Other covariates

Covariate data on maternal and child characteristics were collected in the CANDLE and GAPPS studies via questionnaires and medical record abstraction and harmonized by the ECHO-PATHWAYS consortium. Supplementary Fig. 1 depicts a Directed Acyclic Graph (DAG) for our conceptual model of associations between maternal stress exposures and the placental transcriptome. Confounders were defined as factors that may influence both the exposure and the placental transcriptome, including self-reported race (White vs. Black vs. Multiple race vs. Asian vs. Other vs. American Indian/Alaska Native), self-reported ethnicity (Hispanic/Latino vs. Not Hispanic/Latino), and socioeconomic position. We did not consider race/ethnicity as proxies for genetic ancestry labels. Race is a political and social construct that often serves as a proxy for the impact of racist practices and structural inequality; it is not a biological construct [48] and thus is examined in the current paper with this premise in mind. Models adjusted for multiple covariates that capture different aspects of socioeconomic position, including household income adjusted for region and inflation (continuous), household size (2–3 vs. 4 vs. 5 vs. ≥6), maternal education ( < High School vs. High School completion/Graduate Equivalency Diploma vs. Graduated college or technical school vs. Some graduate work or graduate/professional degree), and geospatially-linked indicators of neighborhood deprivation (continuous standardized score) [49].

We also included precision variables, defined as variables that could affect the placental transcriptome but have no clear casual effect on the exposure. Precision variables included fetal sex (Male vs. Female), maternal gravidity (continuous number of pregnancies), delivery mode (Vaginal vs. C-section), labor type (Spontaneous vs. Spontaneous, augmented vs. Induced vs. No labor), study site, maternal age (continuous years), and sequencing batch. While socioeconomic position variables were conceptualized as confounders in prenatal SLE models, they were measured temporally after maternal childhood, and thus cannot be upstream causes of maternal CTEs. However, socioeconomic position variables may still influence the placental transcriptome and serve as precision variables in maternal CTE exposure models. Alternatively, these variables may still be conceptualized as confounders of the association between maternal CTEs and the placental transcriptome if they serve as proxies for maternal socioeconomic position during childhood.

Finally, we also considered variables that could be potential confounders but may also serve as mediators (i.e., variables on the causal pathway between maternal stress and placental gene expression). These factors included gestational age at birth (continuous weeks), maternal pre-pregnancy BMI (continuous kg/m^2^), and maternal tobacco (Yes vs. No) and alcohol (Yes vs. No) use during pregnancy. Given that BMI, tobacco use, and alcohol use may reflect mechanisms of coping with stress, we considered the models adjusting for these potential mediators as our primary analysis, with the objective of isolating placental transcriptomic responses to maternal stress that are independent of mechanisms of coping with stress. Gestational age at birth was not included as a covariate in the main models because: 1) as opposed to stress-coping pathways, gestational age at birth may reflect potential biological pathways through which maternal stress impacts the placental transcriptome, and 2) it is plausible that placental gene expression is an upstream cause of gestational age at birth, making gestational age a collider rather than a mediator. Thus, gestational age was included as an additional covariate in sensitivity analyses but not the main models. Since including potential mediators is a conservative approach – adjusting for effects that are likely on the causal path between the exposure and placental transcriptome is expected to attenuate results toward the null – we also report sensitivity models that do not adjust for any of these potential mediators. Maternal alcohol use was based on self-report. The positive tobacco exposure group included individuals with maternal urine cotinine above 200 ng/mL [50], as well as individuals who were below this cut-off but self-reported tobacco use during pregnancy. Covariates were determined a priori.

### Statistical analysis

Descriptive statistics were calculated to understand characteristics of the study sample, and to compare our study sample with the entire CANDLE and GAPPS cohort populations. Because prior work in this sample indicates that prenatal SLEs and maternal CTEs may interact in their effects on child health [10], we initially tested associations of SLEs, CTEs, and SLE by CTE interactions with gene expression controlling for all covariates discussed above. There were no significant SLE by CTE interaction product terms for any genes (not shown). Therefore, we constructed separate models to evaluate associations of prenatal SLEs and maternal CTEs with placental gene expression. For all models, independent variables included one of the stress-related exposures along with all covariates discussed above, with gene expression of individual genes acting as the dependent variable. We retained only protein-coding genes, processed pseudogenes, and lncRNAs, and removed genes with average log counts per million (CPM) < 0. Transcript filtering was applied separately to the analytic samples with SLE (*n* = 874) and CTE (*n* = 1 029) data available, resulting in a final sample of 14 047 and 14 030 transcripts for prenatal SLEs and maternal CTEs, respectively. Log-cpm values were normalized to library size using the weighted trimmed mean of M-values [51].

In our primary analysis, missing covariate data were imputed using a multiple imputation method designed specifically for RNA-seq studies, implemented within the RNAseqCovarImpute package [52]. Exposure (stress) and outcome (gene expression) data were not imputed. Unlike single imputation, multiple imputation accounts for uncertainty in the prediction of missing data points. However, because multiple imputation methods must include the outcome as a predictor of missing data to avoid bias [53], they are difficult to apply given the high dimensionality of the outcome data in RNA-sequencing studies (e.g., 14 047 outcome genes). RNAseqCovarImpute accommodates high dimensional expression data by binning genes into smaller groups, creating separate multiply imputed datasets and differential expression models within each bin, and pooling results with Rubin’s rules. This method integrates with the limma-voom differential expression pipeline to fit weighted linear models for each gene that take into account individual-level precision weights based on the mean-variance trend [54]. Results are further moderated using the limma empirical Bayes procedure in which gene-wise variances are squeezed towards a global gene variance prior [55]. We report log_2_-adjusted fold-changes (Log2FCs) for each one number increase in SLEs or CTEs. Log2FCs were transformed into percent gene expression changes using the formula (2^Log2FC^-1)*100%.

For sensitivity analyses, we constructed 1) complete-case analyses, and 2) random forest single imputation models using the missForest package [56]. As in the primary analysis, both sensitivity analyses were fit using the limma-voom pipeline. The voomLmFit function from the edgeR package was used to fit weighted linear models for each gene, and the eBayes function from the limma package was used to apply the empirical Bayes procedure. In all differential expression analyses, genes were considered statistically significant at false discovery rate (FDR) < 0.05 using the Benjamini-Hochberg method [57], and we additionally report the number of genes with FDR < 0.10. We evaluated differences between these two sensitivity analyses and the multiple imputation models by assigning P-value rankings to each gene and comparing these ranks across the three methods.

To identify gene sets and pathways associated with prenatal SLEs and maternal CTEs, we performed Ensemble of Gene Set Enrichment Analyses (EGSEA), an approach that integrates the results of several separate gene set enrichment methods to produce combined P-values and gene set rankings [58]. Specifically, we examined MSigDB hallmark gene sets [59, 60] and KEGG pathways (excluding KEGG human diseases) [61] with the over-representation analysis (ora) [62], globaltest [63], plage [64], safe [65], zscore [66], ssgsea [67], roast [68], fry [68], padog [69], camera [70], and gsva [70] methods. Of note, the compatibility of pathway and gene set enrichment methods with multiple imputation depends on their inputs. The RNAseqCovarImpute multiple imputation method produces one final list of genes with their associated t-statistics, log fold changes, and *P*-values for differential expression. Thus, the method is compatible with gene set enrichment analyses that utilize gene rankings such as ora, or gene level statistics such as camera and gage [71]. However, RNAseqCovarImpute is not compatible with gene set enrichment analyses that require as input a gene expression matrix or data at the individual sample level, as the nature of multiple imputation requires the creation of multiple gene expression matrices across each imputed dataset.

Owing to the limited compatibility of multiple imputation with existing gene set enrichment methods, we utilized EGSEA by passing the “voom” object from the single imputation analysis, which includes the design matrix, precision weights, and normalized log-CPM values, into the egsea function from the EGSEA package. EGSEA was run using all gene set enrichment methods listed above. The single imputation results were considered as a reasonable alternative to the multiple imputation results because, as shown in the results section, the gene P-value rank orders produced by each method were very similar. In an additional sensitivity analysis, we performed gene set enrichment analysis using the gene t-statistics from the multiple imputation differential expression analysis with three compatible methods, namely, ora, gage, and camera, using the EGSEA, gage, and limma packages, respectively.

To explore sex-specific effects, we performed EGSEA separately on male and female strata with the single imputation data as described above.

## Results

Covariate and stress data are described in Table 1. Women reported an average of 1.6 stressful life events (SLEs) during pregnancy (range = 0-14), and 0.5 childhood traumatic events (CTEs) in their own childhood (range = 0-3). Among the participants, 26.1% of women reported exposure to both prenatal SLEs and maternal CTEs, 36.0% prenatal SLEs only, 27.7% neither prenatal SLEs or maternal CTEs, and 10.2% maternal CTEs only. Rates of individual maternal CTEs were as follows: 26.3% witnessed family violence, 8% experienced physical abuse, and 19% experienced sexual abuse. Prenatal SLEs and maternal CTEs were weakly, positively correlated (Spearman’s rho = 0.226). On average, women were 27.9 years of age at delivery (range = 16-43), and most individuals self-identified as White (48.2%) or Black (42.1%). The majority of participants reported no tobacco (92.3%) or alcohol (90.6%) use during pregnancy, underwent labor (83.4%), and delivered vaginally (61.7%). Compared with the entire CANDLE and GAPPS cohorts, our sample with placental transcriptomics data and no placental abruption had higher mean maternal age (27.9 vs. 26.3), lower frequency of prenatal tobacco exposure (7.7% vs. 10.4%), and higher frequency of induced labor (32.5% vs. 27.2%). Socioeconomic status differences were mixed, with our sample having lower mean household income ($56 300 vs. $64 000), but higher maternal educational attainment and lower neighborhood deprivation index (Supplementary Table 2).

**Table 1:** Characteristics of the study sample (*n* = 1 065).

**Prenatal SLEs (sum)**	
Mean (SD)	1.576 (1.861)
Range	0 - 14
Missing	191 (17.9%)
**Maternal CTEs (sum)**	
Mean (SD)	0.533 (0.799)
Range	0 - 3
Missing	36 (3.4%)
**Fetal biological sex (n (%))**	
Male	531 (49.9%)
Female	534 (50.1%)
**Maternal age (years)**	
Mean (SD)	27.884 (5.677)
Range	16 - 43
Missing	20 (1.9%)
**Maternal race (n (%))**	
White	513 (48.2%)
Black	448 (42.1%)
Multiple Race	61 (5.7%)
Asian	17 (1.6%)
Other	11 (1.0%)
American Indian/Alaska Native	<5 ( < 0.5%)
Missing	13 (1.2%)
**Maternal ethnicity (n (%))**	
Not Hispanic/Latino	1000 (94.1%)
Hispanic/Latino	63 (5.9%)
Missing	2 (0.2%)
**Maternal education (n (%))**	
<High School	76 (7.1%)
High School completion	421 (39.6%)
Graduated college or technical school	383 (36.0%)
Some graduate work or graduate/professional degree	183 (17.2%)
Missing	2 (0.2%)
**Family income (USD adjusted for region and inflation)**	
Mean (SD)	56277 (43074)
Range	2493 - 214975
Missing	56 (5.3%)
**Neighborhood deprivation index (standardized score)**^**a**^	
Mean (SD)	0.171 (0.802)
Range	-1.482 - 2.804
Missing	49 (4.6%)
**Pre-pregnancy BMI (kg/m**^**2**^**)**	
Mean (SD)	27.7 (7.5)
Range	14.0 - 62.0
Missing	22 (2.1%)
**Household size (n (%))**	
2-3	208 (20.5%)
4	399 (39.2%)
5	240 (23.6%)
≥ 6	170 (16.7%)
Missing	48 (4.5%)
**Maternal tobacco (n (%))**	
No	982 (92.3%)
Yes	82 (7.7%)
Missing	1 (0.1%)
**Maternal alcohol (n (%))**	
No	958 (90.6%)
Yes	99 (9.4%)
Missing	8 (0.8%)
**Gravidity (number of pregnancies)**	
Mean (SD)	2.6 (1.6)
Range	0.0 - 12.0
Missing	8 (0.8%)
**Labor type (n (%))**	
Spontaneous	241 (23.0%)
Spontaneous, augmented	293 (27.9%)
Induced	341 (32.5%)
No Labor	174 (16.6%)
Missing	16 (1.5%)
**Delivery method (n (%))**	
Vaginal	657 (61.7%)
C-section	408 (38.3%)

^a^Higher value indicates higher deprivation.

**Fig. 2:**
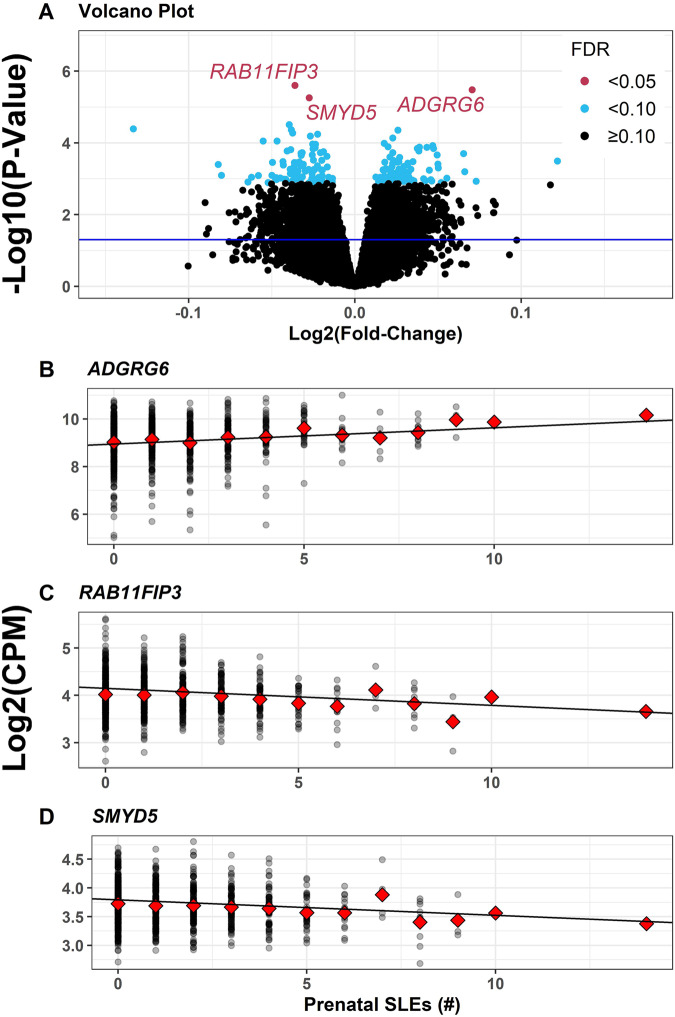
Associations of prenatal SLEs with placental gene expression. Volcano plot depicts log_2_-adjusted fold-changes in 14 047 genes for each one number increase in prenatal stressful life events (SLEs), from multiple imputation analyses adjusting for maternal age, race, ethnicity, pre-pregnancy BMI, gravidity, tobacco and alcohol use during pregnancy, household income adjusted for region and inflation, household size, maternal education, neighborhood deprivation index, fetal sex, labor type, delivery method, study site, and sequencing batch (**A**). Horizontal line at *P* = 0.05. Limma-voom linear model trends (lines) for *ADGRG6* (**B**), *RAB11FIP3* (**C**), and *SMYD5* (**D**). For each gene, one point per participant (gray points) and means (red diamonds) summarize the distributions of log2-counts per million (CPM) normalized to library size at each level of prenatal SLEs.

In placental transcriptome-wide analysis with FDR < 0.05, controlling for potential confounders, mediators, and precision variables, prenatal SLEs were associated with three differentially expressed genes (DEGs) in the multiple imputation models (*n* = 874, Fig. 2A, Supplementary Table 3). Each one number higher prenatal SLE was associated with 5.0% higher expression of adhesion G protein-coupled receptor G6 (*ADGRG6*) (Log2FC = 0.07, Fig. 2B), 2.7% lower expression of RAB11 family interacting protein 3 (*RAB11FIP3*) (Log2FC = -0.04, Fig. 2C), and 2.1% lower expression of SMYD family member 5 (*SMYD5*) (Log2FC = -0.03, Fig. 2D). Results were the same in a sensitivity analysis that additionally adjusted for gestational age at birth (Supplementary Table 3). Results were similar in a sensitivity analysis that did not include potential mediators as covariates, except the association with decreased *SMYD5* expression was no longer significant (FDR *P*-value = 0.074, Supplementary Table 3). At the FDR < 0.10 threshold, prenatal SLEs were additionally associated with upregulation of 89 genes and downregulation of 91 genes (Fig. 2A, Supplementary Table 3).

Controlling for potential confounders, mediators, and precision variables, maternal CTEs were not associated with any DEGs (Supplementary Table 4). Similarly, there were no significant associations of maternal CTEs with gene expression in a sensitivity analysis that additionally adjusted for gestational age at birth and a sensitivity analysis that did not include potential mediators as covariates (Supplementary Table 4). Differential expression analysis results for SLEs and CTEs were similar when using two alternative methods for handling missing data: single imputation and complete case analysis (Supplementary Results, Supplementary Fig. 2, Supplementary Tables 3–6).

**Fig. 3:**
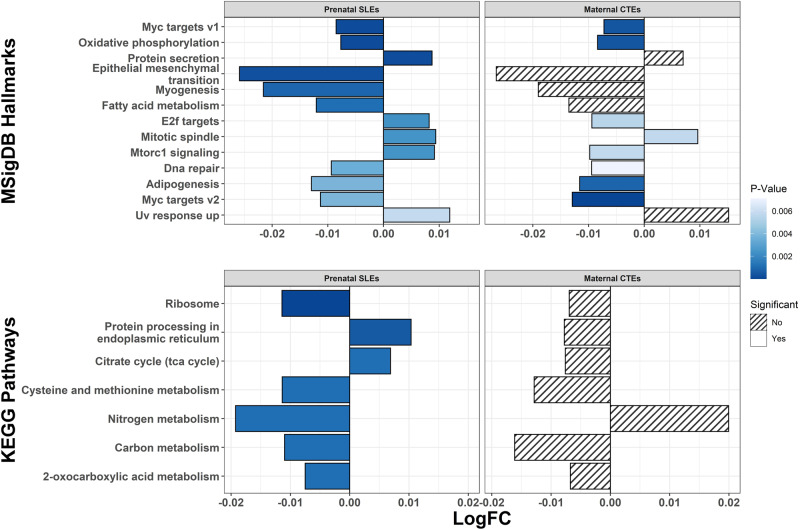
Ensemble of Gene Set Enrichment Analyses for Prenatal SLEs and Maternal CTEs. Associations of mutation signatures database (MSigDB) hallmarks and Kyoto Encyclopedia of Genes and Genomes (KEGG) pathways with prenatal SLEs and maternal CTEs. Log_2_ adjusted fold-changes (LogFC) and P-values come from ensemble of gene set enrichment analyses combining across 11 popular gene set testing methods. All terms with false discovery rate (FDR) adjusted P-value < 0.05 in association with prenatal SLEs and/or maternal CTEs shown.

In addition to evaluating individual gene associations with maternal stress, we performed gene set enrichment analysis using EGSEA on the single imputation results to combine across 11 popular gene set testing methods. All MsigDB hallmark and KEGG pathway terms that were significantly associated (FDR < 0.05) with either prenatal SLEs or maternal CTEs in EGSEA analyses are presented in Fig. 3. Prenatal SLEs were associated with altered regulation of 13 MsigDB hallmark gene sets and 7 KEGG pathways. Although maternal CTEs were not associated with differential expression of any single gene (Supplementary Table 4), they were associated with 8 MsigDB hallmark gene sets (Fig. 3). Among these 8 CTE-associated gene sets, 6 were also significantly associated with prenatal SLEs and in the same direction as their association with maternal CTEs. No KEGG pathways were significantly associated with maternal CTEs (Fig. 3). The EGSEA results on the single imputation models were similar to our sensitivity analyses applying the camera, gage, and ora methods to the multiple imputation differential expression models. As in EGSEA, prenatal SLEs were associated with genes involved in upregulation of the protein secretion and protein processing in the endoplasmic reticulum pathways, and downregulation of the epithelial mesenchymal transition, MYC targets v2, and ribosome pathways (cf. Figure 3, Supplementary Table 7). As in EGSEA, maternal CTEs were associated with downregulation of genes that were members of the MYC targets v1 and v2 pathways (cf. Fig. 3, Supplementary Table 7).

**Fig. 4:**
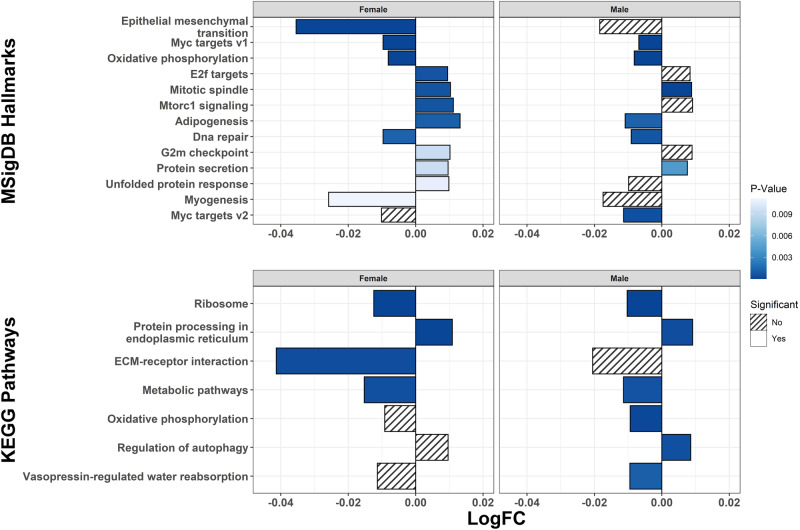
Ensemble of Gene Set Enrichment Analyses for Prenatal SLEs Stratified by Sex. Associations of mutation signatures database (MSigDB) hallmarks and Kyoto Encyclopedia of Genes and Genomes (KEGG) pathways with prenatal SLEs stratified by sex. Log_2_ adjusted fold-changes (LogFC) and P-values come from ensemble of gene set enrichment analyses combining across 11 popular gene set testing methods. All terms with false discovery rate (FDR) adjusted P-value < 0.05 in either stratum shown.

We also performed sex-stratified EGSEA analyses to explore sex-specific effects of maternal stress. All MsigDB hallmarks and KEGG pathways that were significantly associated with prenatal SLEs in either male or female strata are presented in Fig. 4. The majority of gene sets associated with prenatal SLEs were altered in the same direction in both males and females. Among 9 gene sets with significant associations in both strata, 8 were directionally concordant. However, genes related to the adipogenesis pathway were upregulated by prenatal SLEs in females but downregulated in males (Fig. 4).

**Fig. 5:**
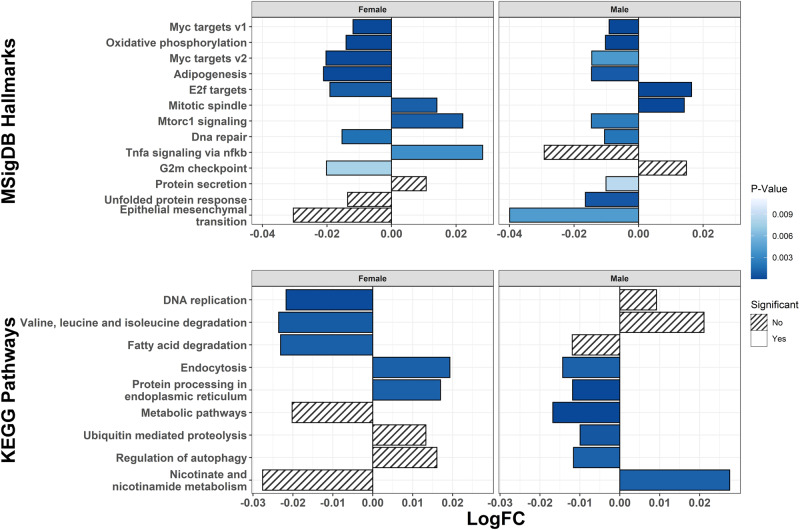
Ensemble of Gene Set Enrichment Analyses for Maternal CTEs Stratified by Sex. Associations of mutation signatures database (MSigDB) hallmarks and Kyoto Encyclopedia of Genes and Genomes (KEGG) pathways with prenatal SLEs stratified by sex. Log_2_ adjusted fold-changes (LogFC) and P-values come from ensemble of gene set enrichment analyses combining across 11 popular gene set testing methods. All terms with false discovery rate (FDR) adjusted P-value < 0.05 in either stratum shown.

All MsigDB hallmarks and KEGG pathways that were significantly associated with maternal CTEs in either male or female strata are presented in Fig. 5. Unlike the similarities across strata for prenatal SLEs, 4 of the 10 gene sets with significant associations in both strata were regulated in opposite directions by maternal CTEs in males and females. Moreover, maternal CTEs were not associated with any KEGG pathways in the main analysis (Fig. 3), but were associated with several KEGG pathways in the sex-stratified analyses, and the directions of association were generally opposite in males versus females (Fig. 5).

## Discussion

In this study of over 1 000 mother-child dyads, maternal stressful life events (SLEs) during pregnancy and childhood traumatic events (CTEs) were associated with gene expression patterns in the placenta. Prenatal SLEs were associated with differences in placental expression of three genes. Both prenatal SLEs and maternal CTEs were associated with transcriptomic differences in pathways from the molecular signatures database (MsigDB) and Kyoto Encyclopedia of Genes and Genomes (KEGG) gene sets, suggesting alterations to placental function, with implications for the etiology of pregnancy complications and adverse birth outcomes.

In our transcriptome-wide analysis, prenatal SLEs were positively associated with *ADGRG6* expression and negatively associated with expression of *RAB11FIP3* and *SMYD5*. *ADGRG6* (also known as *GPR126*) encodes a G protein-coupled receptor and is involved in cellular adhesion. It is expressed specifically within the liver and placenta based on definitions provided by the human protein atlas [72]. In humans, genetic mutations in *ADGRG6* are associated with the most common pediatric skeletal disease, adolescent idiopathic scoliosis [73], and with lethal arthrogryposis multiplex congenita [74]. Recent work using mouse and zebrafish models show that *ADGRG6* inactivation is embryonically lethal owing to defective placental remodeling, which may precede the adverse human health conditions above [75]. *RAB11FIP3* encodes a protein that interacts with the RAB11 family of proteins, which play roles in secretory pathways and protein transport. *RAB11FIP3* is specifically involved in endocytosis and endocytic recycling [76], processes that mediate placental transfer [77]. *SMYD5* is a histone methyltransferase, and has been shown to be involved in epigenetic transcriptional repression of inflammatory response genes [78]. These genes are involved in cellular adhesion, secretion, and inflammation, which are central pathways to core placental functions including maternal-fetal communication and immune protection.

In addition to being the largest analysis of the relationship between prenatal stress and the placental transcriptome to date, this study is also the first to examine the association of maternal preconceptional stress with the human transcriptome. Preconceptional stress, operationalized as maternal CTEs, was not associated with differential expression of any single genes. Compared with prenatal stress, there is a longer latency between maternal childhood trauma and the measurement of the placental transcriptome, which may explain the lack of any CTE-gene expression associations. Additionally, the 3-item CTE measure, which evaluates exposure to violence, physical abuse, and sexual abuse, may capture a different and smaller range of stressful exposures compared with the 14-item prenatal SLE measure, which evaluates a broad range of economic and interpersonal adversities. Similar results were found in a prior ECHO-PATHWAYS study, where higher prenatal SLEs predicted higher levels of child anxiety and depression at age 8-9, while no significant associations were observed for maternal CTEs [11]. Despite the lack of single gene associations, functional enrichment analyses considering the placental transcriptome holistically, rather than as isolated genes detached from biological context, uncovered associations of maternal CTEs with gene sets related to several biological pathways (discussed below).

To the best of our knowledge, this is the first large-scale analysis of prenatal stress and the placental transcriptome that is not linked to natural disasters. Although a prior placental transcriptomic study in 131 individuals documented many differentially expressed genes related to being pregnant during Hurricane Sandy [25], those alterations could be specific to that natural disaster or population, and may not be generalizable to all forms of stress exposure. Moreover, certain differentially expressed genes could be attributable to month/season/year of birth [79, 80], as the control group consisted of children whose mothers were pregnant within a three-year window surrounding the storm. One strength of our study was the adjustment for numerous potential confounders, and socioeconomic confounding either by family income or neighborhood deprivation could explain some of the differences between our results and the differentially expressed genes associated with pregnancy during Hurricane Sandy. Strikingly, however, our results corroborate some of the transcriptomic differences uncovered in that study: *ADGRG6* was upregulated (P-adj = 0.006), and there was evidence that *RAB11FIP3* was downregulated (P-adj = 0.062) in the placentas of participants who were pregnant during Hurricane Sandy versus pregnancies that occurred before Hurricane Sandy [25].

Consistent with prior work implicating perceived stress during pregnancy as a risk factor for preeclampsia [81–83] and reduced birth weight [3, 4], we found that prenatal SLEs were associated with gene expression differences that may increase risk for pregnancy complications and adverse birth outcomes. For instance, prenatal SLEs were associated with several pathways related to protein homeostasis, and the directional changes of these associations were consistent with endoplasmic reticulum (ER) stress. ER stress, which occurs following an imbalance in redox homeostasis between the ER and cytosol, triggers the unfolded protein response and activates signaling events consistent with the suppression of mRNA translational initiation, the upregulation of protein processing in the ER, and the degradation of misfolded proteins [84–86]. In agreement with the suppression of mRNA translational initiation associated with ER stress, increased prenatal SLEs were associated with downregulation of the KEGG ribosome pathway and downregulation of several amino acid metabolism pathways. In agreement with upregulation of protein processing and recycling associated with ER stress, prenatal SLEs were associated with upregulation of pathways related to protein processing in the ER and protein secretion, and, in the multiple imputation analysis, upregulation of ubiquitin mediated proteolysis. The ER is essential for protein processing and secretion, and through this role coordinates signaling pathways regulating metabolism, cell proliferation, and cell death by modifying proteins involved in these biological pathways. These ER functions are critical to the placenta’s role in maternal-fetal signaling, as the peptide hormones involved in this signaling are processed by the placental ER. Thus, perturbation of ER function can interfere with placental endocrine regulation of maternal metabolism, which may ultimately restrict nutrient transport to the fetus [87]. Consistent with the core role of the ER in placental biology, prior work implicates ER stress in the etiology of pregnancy complications including intrauterine growth restriction and preeclampsia [87–90]. Thus, findings from our study support ER stress as a candidate mechanism underlying prenatal psychological stress-associated pregnancy complications.

In addition to ER stress, prenatal SLEs and maternal CTEs were associated with placental gene expression within other pathways that have previously been linked with pregnancy complications. For instance, one study has shown that the down regulation of amino acid transport may precede intrauterine growth restriction [91], and we observed associations of maternal stress with down regulation of several amino acid related pathways (cysteine and methionine metabolism, and valine, leucine, and isoleucine degradation). We also observed negative associations between prenatal stress and expression of genes within the epithelial mesenchymal transition pathway. Invasive extravillous trophoblasts, originating from trophoblast cells through epithelial-mesenchymal transition, are critical for normal placental function. Dysregulated epithelial-mesenchymal transition of extravillous trophoblasts may induce defective migration and invasion and disrupt the process of spiral artery remodeling during the first trimester of pregnancy [92]. The importance of the epithelial mesenchymal transition pathway in normal placental development is underscored by prior work showing that it may be downregulated in preeclampsia [93–96]. Prenatal SLEs and maternal CTEs were also associated with repression of the DNA repair pathway. Down regulation of DNA repair pathways in trophoblasts owing to abnormal RNA degradation and miRNA expression may contribute to recurrent pregnancy loss [97]. Both prenatal SLEs and maternal CTEs were associated with downregulation of MYC signaling, which is involved in multiple growth promoting and signal transduction pathways, most frequently studied in the context of cancer [98]. However, there is evidence that aberrant MYC signaling is related to placental pathology as decreased *MYC* expression has been observed in preeclamptic versus normal human placentas [99].

Prenatal SLEs were associated with several pathways relating to energy metabolism, including oxidative phosphorylation, the citrate cycle (TCA cycle), carbon metabolism, and nitrogen metabolism. The citric acid cycle produces the precursor metabolites that are utilized via oxidative phosphorylation to produce adenosine triphosphate (ATP), the primary cellular energy source. The placenta has substantial energy requirements compared to other tissues, and it may regulate its energy metabolism in response to environmental conditions [15] or stress [100]. It has been recently hypothesized that, in some cases, the inability of the placenta to modulate its metabolism under such circumstances may underlie adverse outcomes such as preeclampsia [101]. However, whether these associations of stress with energy metabolism pathways represent a negative effect of the exposure or an adaptive response remains unknown.

Many of the pathway signatures detected here corroborate prior mechanistic studies of psychological stress and mental health disorders. Consistent with our findings, prior work shows positive associations of the unfolded protein response and ER stress with depression and emotional stressors [102–104]. For instance, elevated expression of ER-related genes has been seen among individuals with major depressive and post-traumatic stress disorders [105, 106], and both ER stress and the unfolded protein response are enhanced in animal models of depression [107, 108]. We also uncovered associations of prenatal SLEs and maternal CTEs with down regulated DNA repair pathways. These results are consistent with prior studies that have linked psychological stress with increased levels of DNA damage owing, in part, to impaired DNA checkpoint and repair mechanisms [109, 110].

Gene set enrichment analyses stratified by sex revealed which pathways were either consistently or differentially expressed depending on fetal sex. For instance, prenatal SLEs and maternal CTEs were associated with upregulation of mTORC1 signaling in females. In males, however, prenatal SLEs had no association with mTORC1 signaling, and maternal CTEs were associated with downregulation of this pathway. Mammalian target of rapamycin (*MTOR*) is a central regulator of protein translation, cell growth, and proliferation [111, 112]. *MTOR* regulates amino acid transport in the placenta [113], and studies show associations of *MTOR* inhibition with IUGR, reduced birth weight, and preeclampsia [114–118]. Prior studies have also shown sex-specific changes in *MTOR*, for instance in association with aging in mice [119] and maternal diet quality in humans [120]. Associations of fetal sex with differential pregnancy outcomes [121] and differential survival in preeclampsia [122] have been reported. It is possible that the sex-specific placental gene expression responses to environmental stress observed here may play a role in differential pregnancy and birth outcomes by sex. However, fetal sex did not modify the relationship between placental gene expression and spontaneous preterm birth in a prior analysis [123].

Our results should be considered in the context of several limitations. First, prenatal SLEs and maternal CTEs were assessed retrospectively. However, prior studies have shown substantial consistency in the retrospective report of exposure to traumatic events during childhood [43] and life experiences during pregnancy [44]. Second, gene expression was measured via bulk placental tissue RNA sequencing, which does not capture the differential transcriptomic contributions of the many different cell types found in placental villous tissue. Confounding by cell type heterogeneity is implausible, however, as placental cell type proportions are not an upstream cause of maternal stress. Nevertheless, changes in cell type proportions within the placenta could mediate the associations of maternal stress with gene expression, and mediation analyses to explore this possibility could be the subject of future studies. Third, generalizability of observational study results should always be considered, as exposure-outcome associations may vary between populations with different characteristics. Improving confidence in generalizability of the results, our study included socioeconomically and racially/ethnically diverse participants from two regionally distinct cohorts, and maternal stress exposure rates were comparable with rates in other populations: the mean of 1.58 SLEs during pregnancy reported here was similar to national rates (mean of 1.78 SLEs in one study of >100 000 participants from 31 states [124]), and rates of individual maternal CTEs in the domains of physical abuse, sexual abuse, and witnessing violence were within the range of rates from other studies [125–127]. Fourth, our prenatal SLE and maternal CTE stress exposures capture different multi-domain stressors, but our modeling approach assumes that each type of stress reflected in the questionnaires has the same effect on placental gene expression. Future research utilizing a larger sample size is needed to adequately assess independent associations of different domains of stress with placental gene expression. Finally, although we addressed potential confounding by including a wide range of maternal and socioeconomic factors in our models, residual confounding is always a possibility in observational studies.

In summary, this study stands apart as the largest investigation of the relationship between maternal stressful experiences during pregnancy and the placental transcriptome, and the first placental transcriptomic study of maternal childhood trauma. Prenatal and preconceptional maternal stress were associated with altered expression of single genes and gene expression pathways that are critical to normal placental functioning. Moreover, our results corroborate prior studies that have implicated many of the specific, directional gene and pathway changes detected here in association with psychological stress, mental health, pregnancy complications and adverse birth outcomes. Accordingly, our findings suggest that maternal prenatal and preconceptional stress may alter placental function, illuminating a potential key mechanism for intergenerational effects of stress on pregnancy, birth, and child health outcomes.

### Supplementary information

Supplemental Figures

Supplemental Results

Supplemental Tables

## Data Availability

The data utilized for this study are not publicly available but de-identified data may be available on request, subject to approval by the internal review board and under a formal data use agreement. Contact the corresponding author for more information.

## Code availability

The RNAseqCovarImpute R package is available at 10.18129/B9.bioc.RNAseqCovarImpute. Sample R code used for these analyses is available at https://github.com/brennanhilton/Placental_Transcriptomic_Signatures_of_Prenatal_and_Preconceptional_Maternal_Stress.
