# Supplementary material for: Placental transcriptomic signatures of prenatal and preconceptional maternal stress
Source: Mol Psychiatry. 2024 Jan 11;29(4):1179–91. doi: 10.1038/s41380-023-02403-6 (PMC11176062; doi:10.1038/s41380-023-02403-6)
Supplement: Supplementary file 1 — Supplemental Figures [file 41380_2023_2403_MOESM1_ESM.docx]

Supplemental Figure 1: Directed Acyclic Graph (DAG)


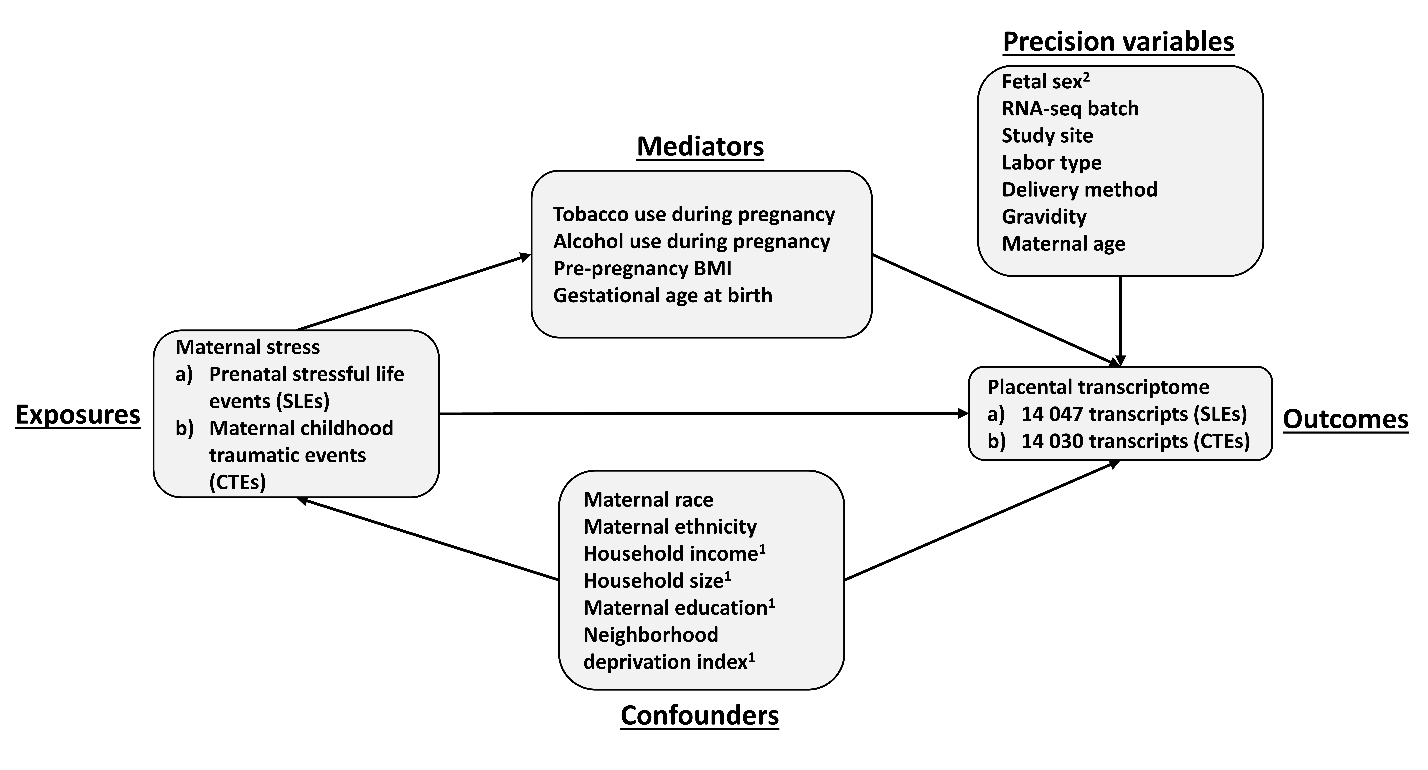


Legend: Directed Acyclic Graph (DAG) depicts conceptual model of association between maternal stress exposures and the placental transcriptome. Confounders are upstream causes of both the predictor and outcome. Mediators are on the causal pathway between the predictor and outcome. Precision variables could affect the outcome but have no clear casual effect on the predictor. The primary analyses adjusted for all variables except for gestational age. A sensitivity analysis additionally adjusted for gestational age. Another sensitivity analysis excluded all potential mediators and only adjusted for confounders and precision variables. Models adjusted for multiple covariates that capture different aspects of socioeconomic position, including household income adjusted for region and inflation, household size, maternal education, and geospatially-linked indicators of neighborhood deprivation.

^1^ While socioeconomic position variables were conceptualized as confounders in prenatal SLE models, they were measured temporally after maternal childhood, and thus cannot be upstream causes of maternal CTEs. However, socioeconomic position variables may still influence the placental transcriptome and serve as precision variables in maternal CTE exposure models. Alternatively, these variables may still be conceptualized as confounders of the association between maternal CTEs and the placental transcriptome if they serve as proxies for maternal socioeconomic position during childhood.

^2^ Primary models adjusted for fetal sex as a precision variable. To explore sex-specific effects, we performed Ensemble of Gene Set Enrichment Analyses (EGSEA) separately on male and female strata.

Supplemental Figure 2: Volcano plots of Prenatal SLEs and placental transcriptome


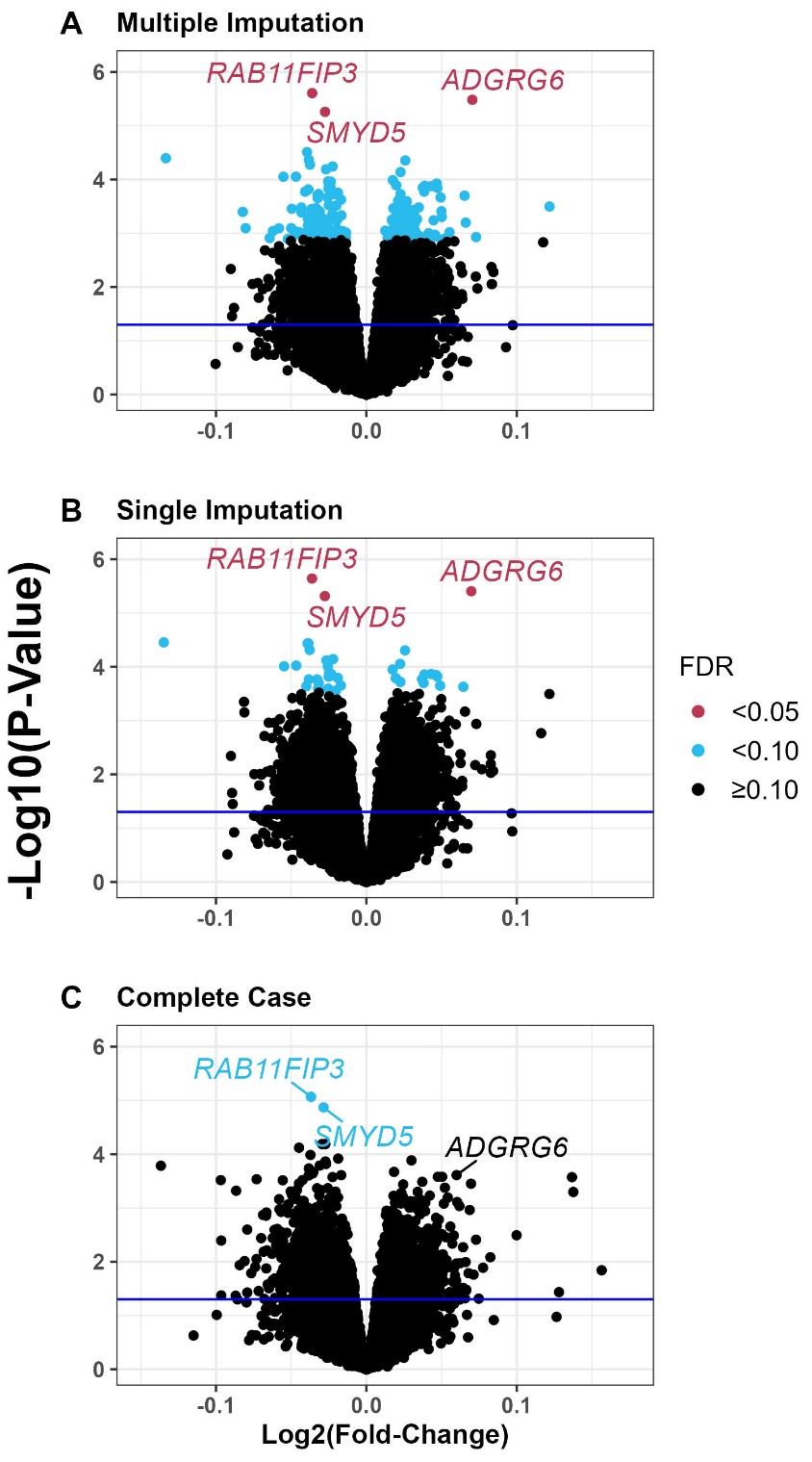


Legend: Volcano plot depicts log_2_-adjusted fold-changes in 14 047 genes for each one count increase in prenatal stressful life events (SLEs) in multiple imputation (A), single imputation (B), and complete case (C) analyses. Models adjusted for maternal age, race, ethnicity, pre-pregnancy BMI, gravidity, tobacco and alcohol use during pregnancy, household income adjusted for region and inflation, household size, maternal education, neighborhood deprivation index, fetal sex, labor type, delivery method, study site, and sequencing batch. Horizontal line at P = 0.05.
