# Supplementary material for: Placental transcriptomic signatures of prenatal and preconceptional maternal stress
Source: Mol Psychiatry. 2024 Jan 11;29(4):1179–91. doi: 10.1038/s41380-023-02403-6 (PMC11176062; doi:10.1038/s41380-023-02403-6)
Supplement: Supplementary file 2 — Supplemental Results [file 41380_2023_2403_MOESM2_ESM.docx]

**Supplemental Results**

*Comparison of differential expression analysis results between multiple imputation, single imputation, and complete case analyses.*

Differential expression results were generally similar across different methods for handling missing data. Prenatal stressful life events (SLEs) were associated with the same three genes in the same directions in both the multiple imputation and single imputation differential expression models (N = 874) – Higher prenatal SLEs were associated with increased expression of adhesion G protein-coupled receptor G6 (*ADGRG6*) and decreased expression of RAB11 family interacting protein 3 (*RAB11FIP3*) and SMYD family member 5 (*SMYD5*) (Supplemental Figure 2, Supplemental Table 3). In complete case analysis (N = 739), although no genes were significant at the FDR<0.05 threshold, higher prenatal SLEs were associated with down-regulation of *RAB11FIP3* (Log2FC = -0.04) and *SMYD5* (Log2FC = -0.03) at the FDR<0.10 threshold (Supplemental Figure 2, Supplemental Table 3).

P-value rankings were similar across all methods for handling missing data (Supplemental Table 5). For example, with slight differences in their order, the top 10 prenatal SLE genes in the single imputation analysis were the same as the top 10 genes in the multiple imputation analysis (*RAB11FIP3*, *ADGRG6*, *SMYD5*, salt inducible kinase 1B (putative) (*SIK1B*), ADAM metallopeptidase domain 15 (*ADAM15*), MIER family member 2 (*MIER2*), transmembrane protein 25 (*TMEM25*), harbinger transposase derived 1 (*HARBI1*), BTB domain containing 2 (*BTBD2*), and aminolevulinate dehydratase (*ALAD*)). Complete case results were also comparable: the top 10 genes in the complete case analysis were all within the top 46 genes in the multiple imputation analysis (Supplemental Table 5).

Across both imputation methods (N = 1 029) and the complete case analysis (N = 861), maternal childhood traumatic events (CTEs) were not associated with expression of any gene (Supplemental Table 4). Although no genes were significant, P-value rankings were similar across all methods for handling missing data (Supplemental Table 6).
